# Phonological complexity, speech style, and individual differences influence ASR performance for Tarifit

**DOI:** 10.1038/s41598-026-43245-w

**Published:** 2026-03-17

**Authors:** Mohamed Afkir, Georgia Zellou

**Affiliations:** Phonetics Lab, Linguistics Department, UC Davis, Davis, CA 95616 USA

**Keywords:** Neuroscience, Psychology, Psychology

## Abstract

This study examines individual differences through the lens of automatic speech recognition (ASR) transfer (applying ASR trained on one language to a new language) from Arabic to Tarifit, an under-resourced Amazigh language with typologically rare phonological structures. Thirty-seven native Tarifit speakers produced target words in both clear and casual speaking styles, allowing us to assess how phonological complexity and speech clarity interact to influence ASR performance. Results show that clear speech significantly improves recognition accuracy, particularly for words with rising sonority onset clusters. In contrast, falling sonority clusters and initial geminate consonants, which are both typologically marked structures, yield higher error rates even when spoken clearly. Importantly, we observe substantial speaker-level variability in ASR outcomes, though demographic factors such as age and gender do not predict performance. These findings suggest that individual differences in speech production and phonological encoding play a critical role in shaping ASR recognition success. By leveraging ASR as a proxy for perceptual processing, this work contributes to our understanding of how phonological structure and speaker variability jointly influence speech perception, with implications for inclusive ASR design and phonological theory.

## Introduction

Automatic speech recognition (ASR) offers a valuable tool for studying speech perception, particularly in the context of typologically rare phonological structures. ASR systems are designed to convert variable acoustic signals into discrete linguistic representations^1^, and their performance often mirrors the perceptual challenges faced by human listeners (e.g^2,3^) Yet, a persistent issue is the “language gap”: ASR systems excel with well-resourced languages but struggle with under-resourced ones that lack annotated corpora and acoustic models^4,5^. The “language gap” refers to the systemic exclusion of non-standard, low-resource, and minoritized language varieties from the datasets used to train ASR and Natural Language Processing (NLP) systems^6,7^. While there are around 7,000 languages worldwide, speech technologies are primarily developed for a small subset of high-resource languages, reinforcing broader inequities^8^. Speakers of underrepresented languages often face misrecognition and exclusion, leading to sociotechnical harms such as accent bias and usability breakdowns^9,10^. One solution is cross-language ASR transfer, in which a system trained on a high-resource language is applied to a low-resource language^11^. The cross-language ASR transfer approach reduces development costs and enables initial performance evaluation without building a system from scratch. However, its success depends on phonological similarity between source and target languages, and it often fails when minority languages exhibit phonological patterns that are rare and underrepresented in majority languages^12,13^.

The present study investigates ASR performance for Tarifit, an under-resourced Amazigh language with typologically rare sound patterns, using a cross-language ASR transfer method (using an ASR system built for Arabic). We specifically ask how phonological complexity, speaking style, and individual variation affect ASR performance for Tarifit which can inform both phonological theory and inclusive ASR design.

## Tarifit: an under-resourced language for ASR

Tarifit (also known as Tarifiyt or Riffian) is an Amazigh (Berber) language spoken primarily in northern Morocco’s Rif region, with notable diaspora communities in Europe. A member of the Northern Amazigh branch, Tarifit features a wide range of syllable types (i.e., CV, VC, CVC, VCC, CVCC, CVVC, and CCVC) and exhibits several typologically rare structures. Notably, it allows complex onsets and initial geminates^14^, as well as onset clusters with not only rising, but also plateauing and falling sonority (e.g.,/ntəf/‘pluck!, /ħkəm/‘judge!, /qtˤəʕ/‘cross the street!’), challenging typological claims that favor rising sonority^15,16^. These features make Tarifit a valuable language for testing phonological theories, particularly those concerning syllable typology and sonority sequencing.

Recent research on ASR performance in related Amazigh languages highlights the theoretical and computational relevance of these phonological features. For example, it has been shown that Kabyle’s gemination and vowel deletion affect ASR accuracy, with orthographic representation (Latin vs. Tifinagh, a script used to write Amazigh languages) playing a key role in recognizing vowels and voiced consonants^17,^^18^. examines ASR transfer from Arabic to Tashlhiyt, revealing systematic transcription errors, especially for vowelless words and casual speech. While clear speech improves recognition, phonotactic mismatches continue to challenge ASR systems. These findings underscore the need for linguistically informed adaptation strategies when extending speech technology to low-resource languages with uncommon phonological structures.

Addressing linguistic disparities in natural language understanding requires linguistically informed approaches to ASR design. Beyond just the social implications, this gap also limits our understanding of how phonological complexity affects recognition performance. Most ASR systems are trained on languages with more typologically common features, leaving languages with rare phonological structures, such as Amazigh languages and varieties, underrepresented. This underrepresentation poses technical challenges for cross-language transfer and highlights the need for empirical studies on phonotactic mismatches in ASR systems. The most frequent syllable type cross-linguistically is the CV syllable, consisting of a single consonant followed by a vowel^19^. This pattern is widely considered unmarked and perceptually optimal. Functional pressures like perceptual clarity, articulatory ease, and learnability are thought to shape the emergence and persistence of such patterns^20,21^. CV syllables also conform to sonority sequencing principles, with the vowel as the sonority peak and the consonant rising toward it, facilitating segmentation and recognition^12,15,16^. How do different types of onset sonority profiles affect ASR performance? Typologically marked structures, such as falling sonority clusters or word-initial geminates, which are common in Tarifit, are hypothesized to be less perceptually salient and harder to process^12,13^, a claim supported by studies showing that human listeners struggle with such contrasts^22,23^. Since ASR errors can reflect listener-like perceptual biases, they provide a computational lens into how phonological complexity affects intelligibility.

Since we are looking at ASR performance for natural speech productions by Tarifit speakers, we also consider how spoken language variation plays a role in cross-language transfer. We ask, does clear speech mitigate errors for marked structures? Clear speech, characterized by hyperarticulation and enhanced acoustic cues, interacts with phonological structure to shape ASR outcomes, suggesting that both human and machine listeners benefit from increased clarity when processing perceptually challenging forms^24,25^. Speakers differ in how they produce and perceive sound structures, influencing which patterns are more robust or vulnerable in communication. Speech directed at machines offers a valuable empirical domain for testing phonological theory, revealing which sound patterns are more resilient under communicative pressure and how speakers adapt their speech. In human-computer interaction (HCI) contexts, users’ mental models of technology shape linguistic behavior, including phonetic accommodation and simplification, reflecting broader principles of linguistic structure and variation^26,27^. Clear speech improves intelligibility for both humans and machines^28–30^, but whether it mitigates errors for typologically rare structures in low-resource languages remains unclear.

In addition to typological complexity and clear speech, individual variation plays a critical role in ASR outcomes. Individual variation in speech production (for instance, differences in articulatory precision, prosodic patterns, and adaptation strategies) is also central to shaping ASR performance^31–33^. Studies of ASR and human-machine interaction often overlook individual variation in phonological behavior. Speakers differ in how they produce and perceive sound structures, influencing which patterns are more robust or vulnerable in communication. Variation in articulatory precision, prosodic timing, and phonological encoding can affect the perceptual salience of a structure^31,34,35^. These speaker-specific patterns are especially relevant in human-computer interaction (HCI), where users adapt their speech to accommodate machine limitations, such as hyper-articulating segments or avoiding complex phonological structures^36,37^. Individual variation is especially salient in languages like Tarifit, where rare phonological structures may be realized differently across speakers due to dialectal variation, multilingual experience, or sociolinguistic background^34,35^. Investigating speaker-level variability provides deeper insight into how phonological patterns are encoded and perceived, and highlights the need for inclusive, speaker-adaptive approaches to speech technology. For low-resource languages, where training data is limited and linguistic diversity is high, accounting for individual variation is essential to improving issues in inclusive technology and phonological theory as well.

### Current study

Building on prior work on cross-language ASR transfer and phonological typology, the present study investigates how phonological complexity and individual variation affect ASR performance for Tarifit, an under-resourced Amazigh language with typologically rare sound patterns. Specifically, we apply an ASR system trained on Arabic to a spoken Tarifit corpus to examine three core questions: (1) Is cross-language ASR transfer from Arabic to Tarifit a viable approach for developing speech recognition for this under-resourced language? (2) How do variations in phonological complexity and speaking style interact to influence ASR performance for Tarifit? (3) Do individual differences across speakers modulate these effects? These questions aim to clarify both the theoretical implications for phonological typology and the practical considerations for inclusive ASR design.

To address these questions, we designed an experiment in which 37 native Tarifit speakers produced target words in two speaking styles (clear and casual) allowing us to assess the role of speech clarity in mitigating recognition errors. The target words included items with typologically marked structures: onset clusters varying in sonority profile (rising, plateauing, falling) and words with initial geminate consonants. These patterns are rare cross-linguistically and absent from Arabic phonotactics, making them ideal for testing how structural complexity interacts with ASR performance.

We use a system trained on Standard Arabic and apply it to our Tarifit corpus. Arabic and Amazigh languages share substantial overlap in their phoneme inventories: both feature a three-vowel system (/a/,/i/,/u/) and a rich set of consonants, including pharyngeals, uvulars, and emphatic segments, which makes it an appropriate test case for cross-language ASR transfer. Yet, the phonotactic profiles of the two languages diverge sharply. Arabic exhibits a strong preference for simple CV syllable structures, generally avoiding complex onsets and codas, and does not permit word-initial geminates or vowelless words. In contrast, Amazigh languages such as Tarifit and Tashlhiyt display high tolerance for marked syllable patterns, including complex onset clusters with falling or plateauing sonority and even vowelless words. They also allow word-initial geminates, a structure absent in Arabic and rare cross-linguistically. These phonotactic differences, despite segmental similarities, potentially pose significant challenges for cross-language ASR transfer, as systems trained on Arabic are not optimized for the structural complexity and typologically rare patterns prevalent in Amazigh languages. Moreover, Tarifit and other Amazigh varieties make heavy use of schwa (sometimes analyzed as a phoneme in Tarifit), which introduces additional ASR challenges because schwa is typically shorter in duration and more spectral variability than full vowels. These are properties that can make it harder for the system to detect and distinguish reliably.

Mixed-effects regression models assessing ASR performance were used to evaluate the effects of speaking style, phonological structure, and speaker-level variability. By analyzing transcription accuracy and edit distance in ASR outputs, we evaluate whether clear speech improves recognition for marked structures and whether certain phonotactic profiles remain challenging even under hyperarticulated conditions. Additionally, we examine speaker-level variability to determine whether individual differences in production contribute to ASR outcomes beyond phonological factors. This approach provides insight into the perceptual salience of rare phonological structures and informs strategies for inclusive ASR design in low-resource contexts.

## Methods

### Target words

We recorded 37 native speakers each producing 80 words in frame sentences in Tarifit. Our word list consisted of items selected for having structures that we examine in the current study:

Word-initial Geminates: Our word list contained 32 items (Table S7 in the Supplementary Materials) that had either word-initial singleton or word-initial geminate consonants. This set of target items was designed to contrast word-initial singleton and geminate consonants in Tarifit. It includes minimal and near-minimal pairs that differ only in the status of the initial consonant, allowing for controlled comparisons of phonological structure. Singleton-initial words (e.g.,/fað/‘thirst’,/qam/‘pre-prayer’) begin with a single consonant, while word-initial geminates (e.g.,/ffam/‘hide’,/qqam/‘read!’) begin with a lengthened consonant segment. The list spans a range of consonants and lexical items, ensuring phonetic diversity while maintaining structural contrast. This design enables investigation into how initial gemination influences acoustic realization and ASR transcription accuracy, particularly in a language where such structures are typologically rare.

Onset Cluster Sonority: This word list comprises 48 Tarifit words (Table S8 in the Supplementary Materials) with complex onset clusters, selected to systematically vary in the sonority relationship between the first and second consonant. Sonority, defined as the relative loudness and resonance of speech sounds, provides a quantifiable measure of acoustic modulation. Each onset cluster was classified as rising, plateauing (matching), or falling, based on the difference in sonority values between the two consonants. Rising clusters (e.g.,/qrib/‘near’) show an increase in sonority from C1 to C2 and are considered unmarked, aligning with preferred syllable structures that peak in the nucleus and taper at the edges^15,16^. Plateauing clusters (e.g.,/sʃən/‘show!, /ħsəb/‘count!’) exhibit minimal sonority change, while falling clusters (e.g.,/ntəf/‘pluck!, /nqər/‘pick!’) show a decrease, and are typologically marked and less frequent across languages^38^. Sonority values were assigned using a universal hierarchy^39^, ranging from 8 for vowels to 1 for voiceless stops. The numeric sonority difference between C1 and C2 was calculated for each word, allowing us to test the prediction that greater sonority rises facilitate ASR recognition, while plateauing or falling profiles pose greater challenges. This design enables a controlled investigation of how phonotactic structure influences ASR performance in a language with typologically rare syllable patterns. The word list also varies in vocalic structure, with some items containing a full vowel and others a schwa in the medial position. This distinction allowed us to examine how vowel quality interacts with onset sonority to influence ASR performance, particularly in a language like Tarifit where schwa plays a central role in syllable formation and morphological structure.

The full set of target words used in the current study is provided in the supplementary materials.

### Speakers

Thirty-seven native Tarifit speakers produced the word list in two speaking styles. Speakers were recorded in Nador, Morocco, and were from Nador. The speakers were divided into two age groups. The younger group comprised 24 individuals (13 male, 11 female) with ages ranging from 18 to 39 and a mean age of 25.79 years (SD = 14.4 years). The older group included 13 individuals (9 male, 4 female), aged 40 to 68, with a mean age of 51.15 years. This demographic spread allowed analysis of age- and gender-related variation in ASR performance across a diverse speaker population. In addition to Tarifit being their first language, all speakers reported speaking Arabic, and most reported speaking at least one other language (French: *n* = 22; English: *n* = 13; Spanish: *n* = 4). Speakers provided informed consent. The study was approved by the UC Davis Institutional Review Board.

### Production

Recordings were made with Audacity in a quiet room using a head‑mounted microphone (Shure WH20XLR) and digitized at a 44.1 kHz sampling rate.

Speakers produced the target words in two different speaking styles: clear and fast speech. For clear speech, we told speakers: “In this condition, speak the words clearly to someone who is having a hard time understanding you.”^40^. Speakers then produced the list in a fast, casual style: “Now, speak the list as if you are talking to a friend or family member you have known for a long time who has no trouble understanding you, and speak quickly”.

The target and filler words were read by speakers in a pseudo-randomized word list. Words were elicited in a frame sentence (*inaji ___ idən:ad*, ‘I say ___ yesterday’). They were presented on a computer screen in Arabic script with the vowels and syllable structure indicated with diacritics (e.g., geminates diacritized with a shadda making consonant gemination, a coda consonant diacritized with a sukun marking no following vowel).

### Speech data processing and coding

First, each speaker’s audio files were hand-segmented into individual target words by a team of trained research assistants and then all segment boundaries were verified by a second researcher (the second author). Then, each audio recording was transcribed using Sonix, an automatic speech recognition (ASR) tool, with the language setting configured to Arabic. The resulting transcripts were downloaded in CSV format, including word-level timestamps. All punctuation was removed from the transcripts prior to analysis. Target words and corresponding ASR output were identified based on matching the timestamps in the audio files and transcripts.

Each target word was assigned a “ground truth” Arabic transcription (non-diacritized, as is the output of the ASR system); these written forms are provided in the Supplementary Materials. We calculated ASR performance in two ways. First, we assessed transcription accuracy (complement of Word Error Rate): Target words were coded for accuracy if the ASR transcription output completely matched the “ground truth” transcription of the word in Arabic script (= 1) or not (= 0). Second, we measured edit distance (also known as Levenshtein distance) for each target word. Edit distance is a quantification of the distance between two strings in terms of substitutions, deletions, and insertions^41^. This measure has been used in linguistic research as a way to quantify phonetic distances between languages and varieties^42^. Edit distance is used here as a measure of relative distance between ground truth and generated transcriptions. Edit distance was computed using standard costs, with substitutions, insertions, and deletions each assigned a cost of 1.

## Results

The data, analysis code, statistical model outputs, and word list for this paper are provided in Open Science Framework (OSF) repository (https://osf.io/g93kt/).

### ASR performance for different types of onset clusters

Our first set of analyses investigated whether ASR performance for words with different onset cluster profiles varies across clear and fast speaking styles.

First, we ran a mixed effects logistic regression model to assess ASR performance in whole word transcription accuracy. The model was run using the *glmer()* function in the *lme4* R package^43^. The model included fixed effects of Speaking Style (clear vs. fast), Onset Cluster Sonority value (centered), and Word Type (CCəC vs. CCVC), as well as two-way interactions between Speech Style and Onset Sonority Value and Speech Style and Word Type. In order to account for variability across speakers and items, we also included by-speaker and by-item random intercepts and by-speaker random slopes for Style, Onset Sonority, Word Type and the interaction between Style and Onset Cluster. Categorical predictors were sum-coded. (Model syntax: Accuracy ~ Style*Onset Cluster Sonority + Style*Word Type + (1 + Style*Onset Cluster+Word Type|Speaker) + (1|Item). Full model output provided in Table S1 in the supplementary materials repository.)

**Fig. 1:**
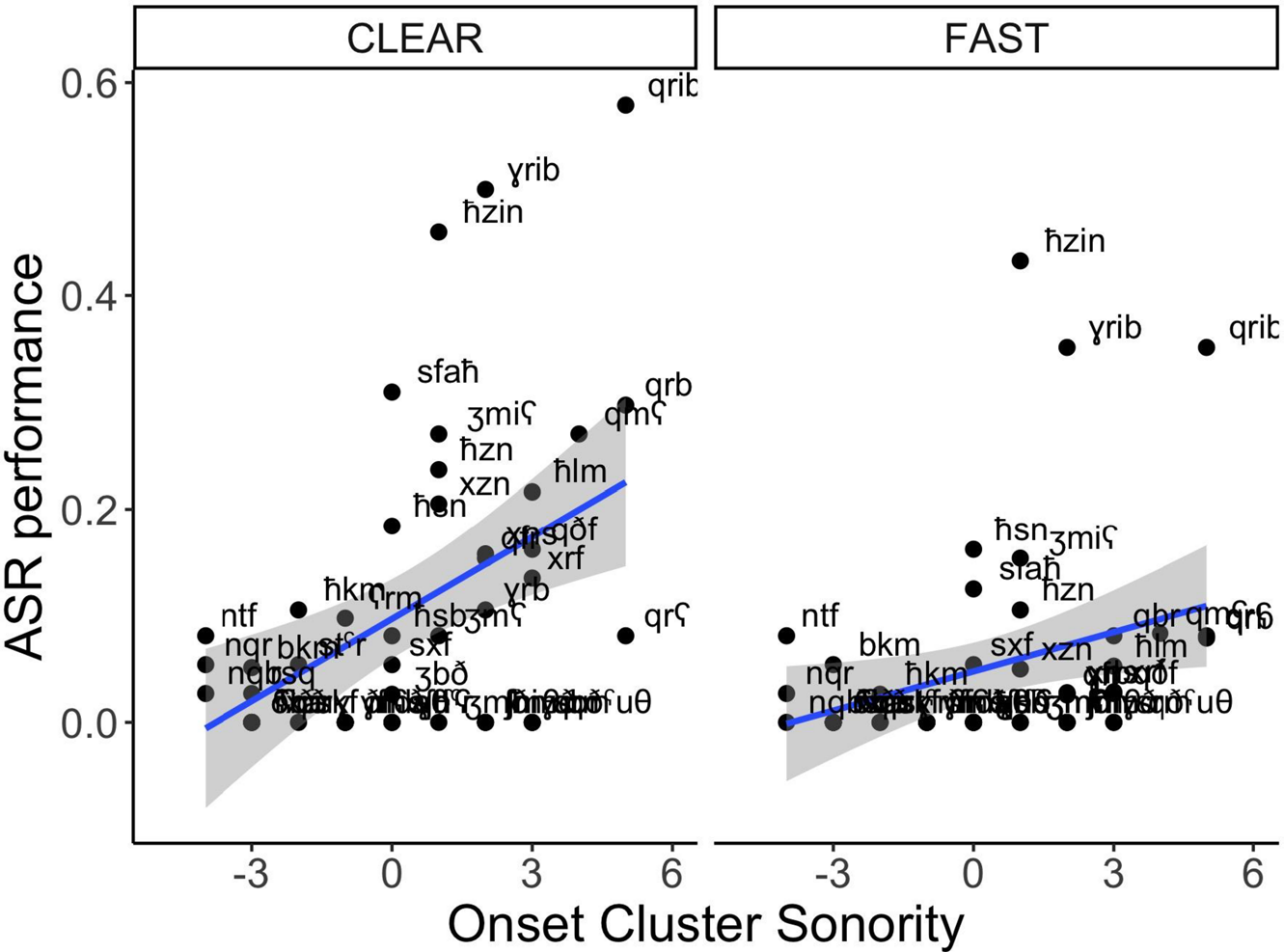
ASR accuracy for words varying in onset cluster sonority profiles (quantified as a numerical value on the x-axis) across clear and fast speaking styles.

Figure 1 plots ASR accuracy for items based on their onset cluster sonority value across speaking styles. The low intercept (Estimate = − 4.25, *p* < 0.01) indicates poor overall recognition of Tarifit. Yet, an effect of Speaking Style indicates that ASR performance improves for clear speech (Estimate = 0.41, *p* < 0.01). There was also an effect of Onset Cluster Sonority (Estimate = 0.83, *p* = 0.02) indicating better ASR performance is associated with words containing rising sonority onset clusters. There was no effect of Word Type, meaning there was no difference in ASR accuracy for CCəC and CCVC words. No interactions were significant.

**Fig. 2:**
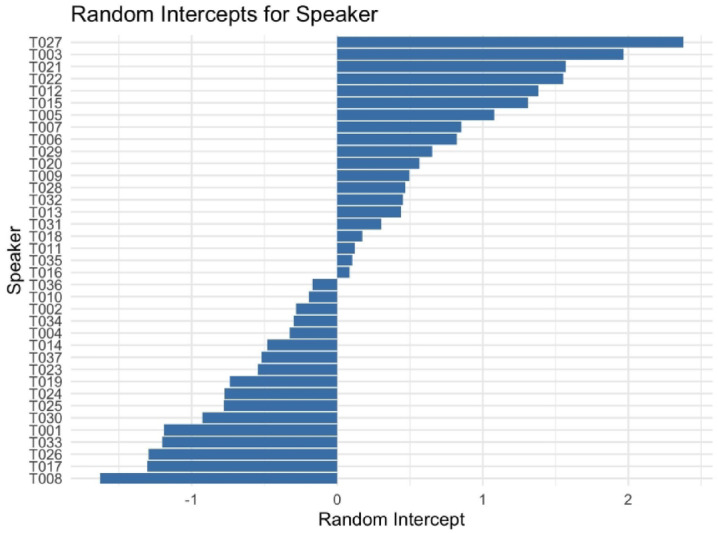
Speaker random intercepts for ASR accuracy.

Random effects showed substantial variability across speakers and lexical items (Speaker variance = 1.3, and Item variance = 3.8). Speaker participant random intercepts from our model are provided in Fig. 2, illustrating the larger cross-speaker variation. Yet, smaller variances for style, onset sonority, and word type indicate modest individual differences in how these factors affect ASR performance.

Next, we ran a mixed effects linear regression model in R to evaluate edit distance from ground truth in the ASR transcription outputs. The model contained the same model structure as that for accuracy (model syntax: Edit Distance (centered) ~ Style*Onset Cluster Sonority + Style*Word Type + (1 + Style*Onset Cluster Word Type|Speaker) + (1|Item), Full model output provided in Table S2 in the supplementary materials repository).

**Fig. 3:**
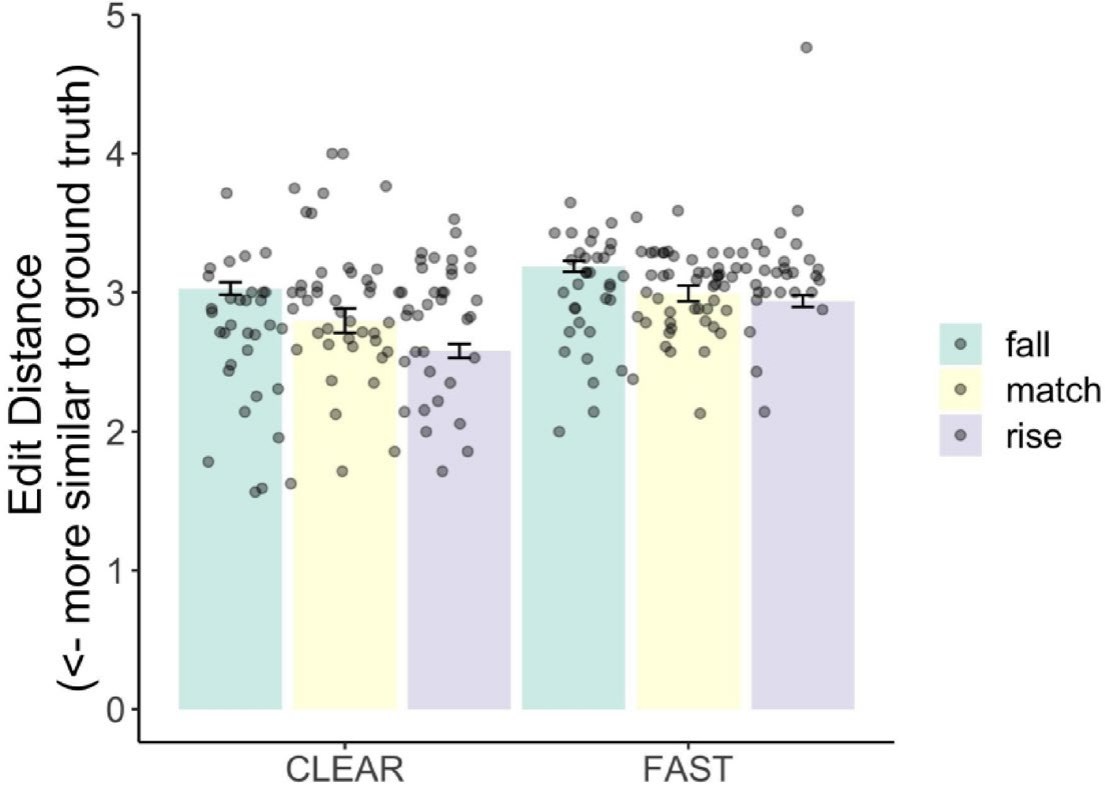
Edit distance in ASR transcriptions for words varying in onset cluster sonority profiles (falling, matching, and rising sonority) across clear and fast speaking styles. Dots represent individual speaker means for each condition.

The model computed an effect of speech style (Estimate = −0.11, *p* < 0.001), wherein clear speech significantly reduces edit distance, meaning ASR performance was closer to ground truth when speech is articulated clearly. There was also an effect of Onset Cluster Sonority (Estimate = −0.12, *p* = 0.03) indicating that there are fewer ASR transcription errors in words containing rising onset clusters. Words with the CCəC structure are associated with lower edit distance (Estimate = −0.17, *p* = 0.01), suggesting they are more accurately transcribed by the Arabic ASR system than CCVC words. There was also an interaction between Style and Onset Cluster Sonority (Estimate = −0.04, *p* = 0.01). This interaction is visualized in Fig. 3, showing edit distance for words with falling, plateauing (matching), and rising onset clusters across clear and fast speech. As seen, the benefit of clear speech on reducing transcription errors is enhanced for words with rising onset clusters. The model also revealed moderate variability across speakers and items (Speaker variance = 0.04, Item variance = 0.12); There were smaller variances for style, sonority, and word type suggesting more consistent effects for these factors across individuals.

We next analyzed categorical error types across cluster types and speech styles. Tokens with no edits were labeled as Exact match (marked as accurate in the above analysis). Tokens with an edit distance greater than 0 were marked as Whole-word error (no overlapping segments between ground truth and ASR output), Substitution (cases involving only substitutions of individual characters), Deletion (deletion of individual characters), Addition (insertion of characters). When multiple edit types occurred within the same token, the error was classified as Mixed edits. This approach allowed us to quantify the types of error patterns across conditions.

Table 1 presents the counts and proportions of error types for each onset cluster sonority profile (falling, matching, and rising sonority) across clear and fast speaking styles.

**Table 1:** Counts and proportions of categorical ASR error types for across onset cluster sonority profiles of words (falling, matching, rising) and speaking styles (clear vs. fast). Each cell reports the count and proportion of tokens within that condition. Error types were derived from character-level edit operations: Exact match indicates no edits (accurate transcription), Whole-word error reflects complete mismatches with no shared segments, Substitution involves only character replacements, Addition and Deletion involve insertion or removal of characters, and Mixed edits include multiple edit types within a token.

Exact match (accurate)	Clear - falling	Clear - matching	Clear - rising	Fast - falling	Fast - matching	Fast - rising
	20 (3.1%)	25 (9.4%)	146 (16.9%)	7 (1.1%)	13 (5.0%)	73 (8.5%)
Substitution	96 (14.9%)	43 (16.2%)	119 (13.8%)	78 (12.2%)	38 (14.5%)	120 (14.0%)
Addition	42 (6.5%)	13 (4.9%)	50 (5.8%)	38 (5.9%)	3 (1.1%)	41 (4.8%)
Deletion	1 (0.2%)	1 (0.4%)	1 (0.1%)	0 (0.0%)	0 (0.0%)	0 (0.0%)
Mixed error	163 (25.3%)	62 (23.4%)	185 (21.4%)	130 (20.3%)	51 (19.5%)	174 (20.3%)
Whole word error	322 (50.0%)	121 (45.7%)	364 (42.1%)	387 (60.5%)	157 (59.9%)	451 (52.5%)

The error-type distribution revealed a strong effect of speech style, with clear speech associated with less extreme error types. Specifically, whole-word errors dominated across conditions but were markedly lower in clear speech (807 tokens; 45.5%) compared to fast speech (995 tokens; 56.5%). Conversely, exact matches were more frequent in clear speech (191 tokens; 10.8%) than in fast speech (93 tokens; 5.3%), indicating that ASR performance was closer to ground truth when articulation was careful.

There was also an effect of onset cluster sonority: words with rising clusters showed the highest proportion of exact matches (146 in clear, 73 in fast) and the fewest whole-word errors relative to falling and plateauing clusters, suggesting that rising clusters are easier for the ASR system to process. Mixed edits and substitutions were common in both styles but slightly more prevalent in clear speech, reflecting that when errors occur under clear articulation, they tend to involve partial segment-level changes rather than complete misrecognition. Overall, the interaction between style and sonority mirrors the regression findings: the benefit of clear speech is amplified for rising clusters, reducing severe errors and increasing transcription accuracy.

### ASR performance for initial geminates

We next analyzed ASR performance, based on accuracy and edit distance in speech transcription, for words containing initial singleton versus geminate onsets.

Our first model evaluated how speech style and onset type influence ASR accuracy. The model contained fixed effects of Speaking Style (clear vs. fast) and Onset Type (C: VC vs. CVC), as well as the two-way interaction between Speech Style and Onset Type. The model also included by-speaker and by-item random intercepts, in addition to by-speaker random slopes for Style and Onset Type and their interaction. Categorical predictors were sum-coded. (Model syntax: Accuracy ~ Style*Onset Type + (1 + Style*Onset Type|Speaker) + (1|Item). Full model output provided in Table S3 in the supplementary materials repository.)

An effect of speech style confirms again that clear speech significantly improves ASR accuracy (Estimate = 0.52, *p* < 0.01). This is consistent with our prediction that more careful speech production improves speech recognition. There was not an effect of Onset type, nor an interaction between Style and Onset Type. Yet, the model did reveal substantial variability across speakers and especially across lexical items (Speaker variance = 0.6 and Item variance = 9.8). Smaller variances for style, onset type, and their interaction suggest a relatively consistent role of these factors across individuals.

Next, we ran a linear mixed effects regression model on edit distance for these words. The model contained a similar structure as the logistic regression for these items. However, we had to simplify the random effects structure to include just random slopes for the main effects by speaker due to overfitting. (Model syntax: Edit Distance (centered) ~ Style*Onset Type + (1 + Style+Onset Type|Speaker) + (1|Item). Full model output provided in Table S4 in the supplementary materials repository.)

**Fig. 4:**
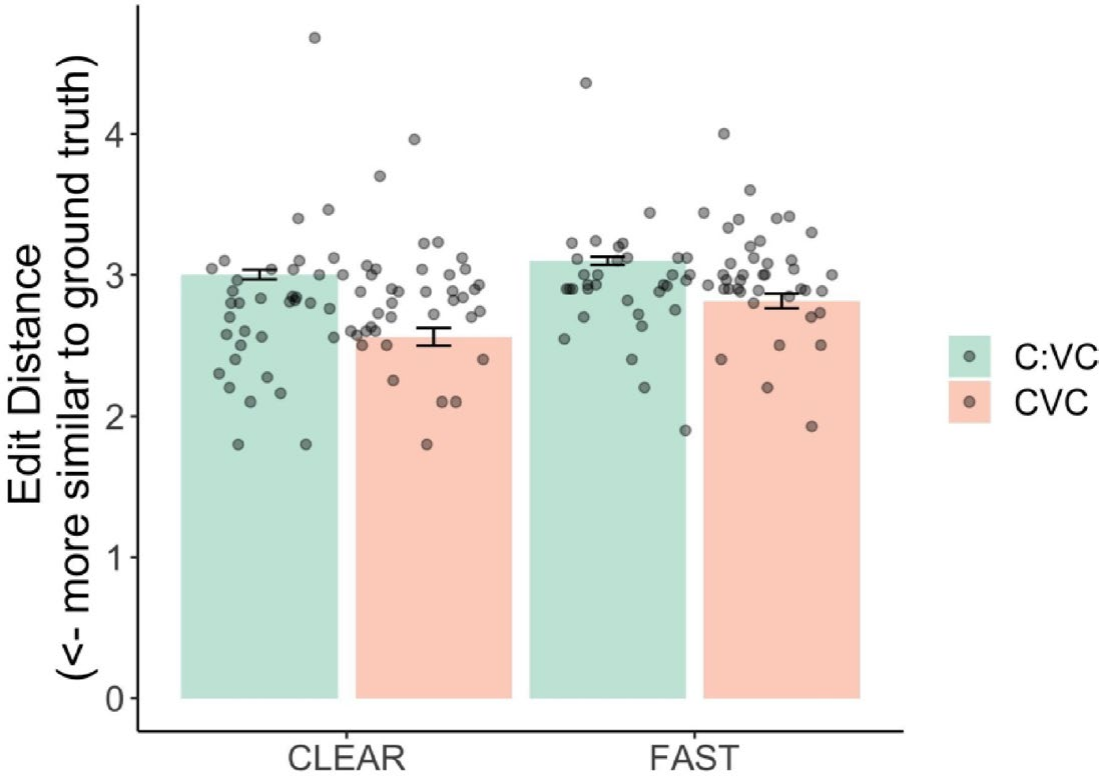
Edit distance in ASR transcriptions for words contrasting in singleton versus geminate onsets across speaking styles. Dots represent individual speaker means for each condition.

Clear speech reduces edit distance (Estimate = −0.09, *p* < 0.01), meaning that the ASR system transcribes clear speech more accurately. There was also an effect of Onset Type: Words with initial geminate consonants are associated with higher edit distances, suggesting they are more difficult for ASR systems to transcribe correctly (Estimate = 0.15, *p* = 0.02). There was also an interaction between Style and Onset type (Estimate = 0.04, *p* = 0.04). This small effect size, illustrated in Fig. 4, indicates that the benefit of clear speech is somewhat reduced for words with initial geminates. In other words, even when spoken clearly, these onset types still pose challenges for the Arabic ASR system. There was moderate variability across speakers and lexical items (Speaker variance = 0.1 and Item variance = 0.1); However, small variances for by-speaker style and onset type slopes suggest consistent effects across individuals.

Table 2 presents the counts and proportions of error types for each onset type (geminate vs. singleton) across clear and fast speaking styles.

The error-type distribution for words with initial geminate (C: VC) versus singleton (CVC) onsets revealed a clear effect of speaking style, with clear speech associated with fewer severe errors overall. Specifically, whole-word errors were the most frequent error type across conditions but occurred less often in clear speech (686 tokens; 50.7%) compared to fast speech (782 tokens; 58.8%). Exact matches were more common in clear speech (77 tokens; 5.7%) than in fast speech (34 tokens; 2.6%), indicating that ASR performance was closer to ground truth when articulation was careful.

There was also a robust effect of onset type: words with initial geminate consonants (C: VC) showed higher proportions of whole-word errors (518 in clear, 582 in fast) and fewer exact matches (38 in clear, 19 in fast) relative to singleton onsets, suggesting that word-initial geminates pose greater challenges for the ASR system. Mixed edits and substitutions were frequent in both onset types, but their relative proportions were slightly higher for geminates, reinforcing the difficulty of these forms even under clear speech.

Finally, the interaction between style and onset type mirrors the regression findings: while clear speech improves accuracy overall, the benefit is attenuated for word-initial geminates, which continue to exhibit elevated error rates compared to singleton words.

**Table 2:** Counts and proportions of categorical ASR error types for words with initial geminate (C: VC) versus singleton (CVC) onsets across clear and fast speaking styles. Each cell reports the number of tokens and percentage within that condition. Error types were derived from character-level edit operations: Exact match indicates accurate transcriptions with no edits, Whole-word error reflects complete mismatches with no shared segments, Substitution involves only character replacements, Addition and Deletion involve insertion or removal of characters, and Mixed edits include multiple edit types within a token.

Exact (accurate)	Clear - geminate	Clear - singleton	Fast - geminate	Fast - singleton
	38 (3.9%)	39 (10.0%)	19 (2.0%)	15 (4.0%)
Substitution	192 (19.9%)	58 (14.9%)	158 (16.6%)	58 (15.4%)
Addition	7 (0.7%)	37 (9.5%)	7 (0.7%)	34 (9.0%)
Deletion	3 (0.3%)	2 (0.5%)	0 (0.0%)	0 (0.0%)
Mixed error	205 (21.3%)	85 (21.9%)	188 (19.7%)	69 (18.4%)
Whole word error	518 (53.8%)	168 (43.2%)	582 (61.0%)	200 (53.2%)

### Post-hoc: exploring the sources of individual differences

Given the large amount of individual variation in ASR transfer performance across the 37 speakers in the present study, we asked if there are systematic social factors that can explain when the speech recognition system is more or less accurate. Prior work on speech recognition performance for high-resource languages has shown that factors such as speaker gender^10,44^ and speaker age^45^ predict ASR transcription differences across talkers. Much of this can be related to disparities that exist in the training data^46^. The Tarifit speakers in the present study varied in these social characteristics, therefore we performed two post-hoc models testing whether systematic differences due to speaker age and gender can explain some of the variance.

First, we ran a logistic regression on ASR accuracy for the entire dataset (target words and filler items) that contained fixed effects of Speaking Style, Speaker Gender (Female vs. Male), Speaker Age group (younger vs. older), and all two- and three-way interactions between these predictors. The random effects structure included by-speaker and by-item random intercepts and by-speaker random slopes for Style. (Model syntax: Accuracy ~ Style*Gender*Age + (1 + Style|Speaker) + (1|Item). Full model output provided in Table S5 in the supplementary materials repository.) The intercept of the model is significantly negative, suggesting low baseline ASR accuracy. Style has a significant positive effect (Estimate = 0.41, *p* < 0.001), indicating that clear speech improves ASR transfer accuracy. Gender, Age, and their interactions with Style are not significant, suggesting that these social factors do not influence ASR accuracy in this dataset. The random effects show substantial variability across speakers and items, especially items (variance = 6.6), indicating that some words are much easier or harder for ASR systems to recognize.

Next, we ran a mixed effect linear regression model on edit distance values for the full set of target and filler items that contained the same model structure as the logistic model: Model syntax: Edit Distance (centered) ~ Style*Gender*Age + (1 + Style|Speaker) + (1|Item) (Full model output provided in Table S6 in the supplementary materials repository.). The model computed an effect of Style (Estimate = −0.9, *p* < 0.001), indicating that clear speech reduces edit distance, i.e., improves ASR transcription accuracy. Neither Speaker Gender nor Age had an effect on edit distance. None of the interactions were significant, suggesting that combinations of these social factors do not meaningfully influence ASR transcription error.

### Interim summary of all results

Across both subsections, clear speech reliably improved recognition (higher accuracy, lower edit distance), with the largest gains for words whose onset clusters had rising sonority; falling and plateauing clusters showed smaller benefits. Initial geminates remained systematically more error‑prone than singleton onsets even under clear speech, which indicates that these typologically marked structures pose persistent challenges for Arabic‑trained ASR in Tarifit. Speaker- and item-level variability was substantial, but did not overturn these patterns.

## General discussion

This study investigated the transfer of automatic speech recognition (ASR) from Arabic to Tarifit, a low-resource and understudied language with no existing commercial ASR system and limited language technology. We found robust effects of speaking style and phonological structure on recognition outcomes. Clear speech improved whole-word accuracy and reduced edit distance across analyses. Phonotactic profiles mattered: rising sonority onset clusters were recognized more accurately than plateauing or falling clusters, and word-initial geminates were consistently harder for the Arabic ASR to transcribe than singleton onsets. While we observed substantial speaker-level variability, demographic factors (age, gender) did not predict performance. There were also substantial item-level differences. Together, these results indicate that structural markedness and clarity jointly shape ASR transfer success in Tarifit and that speaker-specific production characteristics and item-specific variation contribute meaningfully to recognition variability. We discuss each of the findings in turn below, and highlight the importance of individual variation, phonological structure and speech clarity in cross-language ASR applications, especially for languages with minimal technological support.

### Clear speech

Our findings confirm that clear speech in Tarifit significantly improves cross-language ASR transfer from Arabic, enhancing both whole-word accuracy and reducing edit distance compared to casual speech. This pattern is consistent with extensive research showing that clear speech boosts intelligibility across listener populations, including native and non-native speakers, individuals with hearing impairments, and machine-based systems^18,24,28,31^. These results support models in which speakers dynamically modulate phonetic detail to meet communicative demands (e.g^47^) and extend prior work by demonstrating that clarity benefits are structure-dependent: rising sonority clusters gain the most from hyperarticulation, while word-initial geminates remain challenging even when spoken clearly.

In low-resource language contexts like Tarifit, clear speech offers a practical, low-cost strategy for improving ASR performance. Unlike model retraining or data augmentation, speaking clearly is an intuitive adjustment accessible to everyday users. Our error‑type analysis shows that clear speech not only increases accuracy but also shifts the error profile, reducing catastrophic whole-word errors and increasing partial matches (substitution/mixed edits). This suggests that hyperarticulation enhances segmental recoverability even when phonotactic mismatches persist. These findings have direct implications for human-computer interaction: adaptive prompts encouraging clarity could mitigate recognition failures in multilingual settings where ASR systems often struggle due to limited training data (e.g^48^). For ASR developers, these results highlight the need to incorporate speaker-adaptive strategies alongside phonotactic-aware modeling to improve recognition equity in under-resourced languages.

### Disparities across different phonological patterns

Our findings show that falling sonority onset clusters and initial geminates remain major sources of ASR difficulty, even under clear speech conditions. These patterns reflect phonotactic and acoustic mismatches between Tarifit and Arabic: falling clusters, where sonority decreases from C₁ to C₂, are typologically marked and rare across languages^49^, while initial geminates are structurally complex and absent from Arabic phonotactics^50,51^. Such structures are unlikely to appear in commercial ASR training data, making them particularly vulnerable in cross-language transfer.

From a typological perspective, both falling onset clusters and initial geminates are marked structures, which means they are less frequent in universal inventories and acquired later in language development^52^. ASR systems trained on high-resource languages struggle to generalize to these rare forms, especially in zero-shot or low-resource scenarios. Our results reinforce this challenge and highlight the need for phonotactically-aware modeling when extending ASR to underrepresented languages.

Interestingly, the largest disparities emerged in clear speech. While clarity generally improves recognition, it does not uniformly neutralize markedness. Instead, clarity amplifies contrasts between patterns well-represented in the source model and those that are not. This interaction reveals a compounding effect: typologically rare structures (here, falling clusters and geminates) remain error-prone even when hyperarticulated, and become especially problematic in casual speech. ASR systems are thus most vulnerable when both phonological complexity and reduced clarity coincide.

These findings align with phonological typology theories emphasizing the need to fully account for marked structures^49,53^ and that they also are underrepresented in ASR training data. They underscore the need to consider both phonological features and speaking style in cross-language ASR transfer. To improve recognition robustness and equity in low-resource settings, future ASR development must integrate typological diversity, phonotactic constraints, and speaker-adaptive strategies that leverage clarity without assuming uniform benefits across all structures.

### Individual variation

We observed substantial cross-speaker variability in ASR transcription accuracy and edit distance, indicating that individual differences are a key source of variance in cross-language ASR transfer. Yet, the effects of speaking style, syllable sonority, and word type were relatively consistent across individuals, as shown by smaller variances in these predictors. Post-hoc analyses revealed no significant effects of speaker age or gender. This suggests that, in low-resource or cross-language ASR contexts, variation in recognition accuracy is driven primarily by phonological and acoustic mismatches between the source and target languages, rather than demographic factors. In other words, age and gender account for little variance compared to structural and acoustic differences that dominate system performance^10,44^.

The absence of demographic effects does not imply that speaker-level variation is random. Instead, it highlights the importance of cognitive and linguistic factors (e.g., multilingualism, language dominance, and phonological awareness) that may shape ASR outcomes. Multilingual speakers, for instance, often exhibit enhanced metalinguistic awareness and flexible perceptual strategies, which can influence how they produce and perceive phonological contrasts^54,55^. Similarly, greater dominance in the target language or frequent exposure to voice-enabled technologies may improve adaptation to ASR systems^56,57^.

These individual differences offer insight into perceptual encoding strategies and how speakers represent and prioritize phonological information during speech production and recognition. Variation in articulatory precision or prosodic timing may reflect differences in encoding sonority or segmental boundaries, directly impacting ASR accuracy. In low-resource languages like Tarifit, where marked phonological structures are underrepresented in training data, speaker-specific strategies may play an even more critical role. This underscores the need to consider perceptual variation, especially in multilingual and racially diverse populations^56,57^, when designing inclusive ASR systems and language learning tools.

Overall, these findings call for more nuanced models of speaker variability in ASR -- ones that move beyond demographic profiling to incorporate linguistic experience, cognitive flexibility, and phonological encoding preferences. In multilingual and low-resource settings, where typological mismatches and limited training data amplify individual differences, speaker-adaptive strategies are essential. Such approaches not only improve ASR performance but also deepen our understanding of speech perception across diverse populations.

### Implications for inclusive ASR design and phonological theory

Our recommendation does not imply that Arabic ASR systems should simply be expanded to include Tarifit. Rather, our findings highlight that any ASR solution for Tarifit, whether adapted from an existing multilingual model or developed as a dedicated system, must consider factors such as typological variation (e.g., sonority profiles, gemination) and speaking style differences to improve recognition robustness and equity. Cross-language transfer, as used in this study, provides a practical baseline for evaluating performance in low-resource contexts, but it is not a long-term solution. Future development should prioritize multilingual or dedicated models that integrate phonological diversity and speaker-adaptive strategies, rather than relying solely on transfer from typologically mismatched languages.

To achieve this, phonotactically-aware multilingual models are essential. Our results underscore the importance of explicitly modeling sonority sequencing and gemination in ASR systems, either through training data diversification (including languages with marked syllable patterns) or phonologically-informed augmentation that simulates falling/plateauing clusters and initial geminates^58^. In addition, speaker-adaptive prompting can also offer a practical, low-cost intervention: because clear speech reduces catastrophic errors, human-computer interaction strategies that encourage clarity (e.g., adaptive prompts after misrecognition; guidance on slowing or emphasizing clusters) can improve recognition in under-resourced contexts^24,28,59^. Finally, error-aware evaluation should complement traditional metrics like WER. Reporting error-type distributions helps isolate catastrophic versus partial failures and identify where adaptation is most needed (e.g., geminate onset confusion vs. cluster parsing), enabling ASR practitioners to prioritize targeted fine-tuning or rule-based constraints for vulnerable structures.

Beyond applied implications, our findings provide computational evidence for phonological theory. The asymmetries we observe support typological claims linking marked structures to perceptual difficulty^12,13^: rising clusters behave as predicted by sonority-driven accounts^15,16^, while falling clusters and geminates incur greater processing costs. We also identify a clarity–markedness interaction, showing that hyperarticulation enhances recoverability but does not uniformly neutralize marked structures under transfer, refining perspectives on perceptual salience and the H&H framework^60^.

We also find that schwa is not a locus of transfer difficulty. Despite its absence in Arabic’s phoneme inventory, ASR performance did not differ between schwa-containing words and those with full vowels. This indicates that vowel inventory mismatches do not uniformly lead to recognition problems in cross-language ASR transfer. In other words, not all phonological incongruencies are equally problematic: segmental gaps like schwa may be less consequential than phonotactic mismatches such as complex clusters or gemination. This invites theory-driven experiments on why reduced vowels appear perceptually and computationally robust, and how they interact with onset cluster profiles to influence intelligibility in typologically rare syllable systems^61^.

Finally, our study also connects computational modeling to second language (L2) perception research. While our focus is on cross-language ASR transfer for Tarifit, the approach offers a framework for exploring second language (L2) perception. Because ASR systems often exhibit perceptual biases similar to human listeners, they can serve as proxies for modeling how non-native speech is processed. This opens opportunities for future experiments where ASR tools approximate human perception in contexts where recruiting human participants is challenging. Such work could inform theories of L2 phonological acquisition and provide practical insights for language learning technologies.

## Limitations and future directions

Two limitations suggest concrete next steps. First, our transfer used a single Arabic ASR; future work could contrast multiple source models (with varying phonotactic coverage) to separate model-specific from structural effects. Second, we focused on word-level targets; expanding to phrasal/prosodic contexts would probe how boundary cues and speech rate moderate cluster parsing and geminate detection. Finally, given the role of schwa, future studies should stratify items by vocalic structure (full vowel vs. schwa) to quantify its independent and interactive effects on error types.

In sum, this study clarifies where cross-language ASR transfer breaks down for Tarifit (falling clusters, geminates, schwa contexts), how clear speech reshapes failure modes (from whole-word to partial errors), and why item-level phonological complexity eclipses demographic predictors. These insights provide actionable guidance for inclusive ASR, such as phonotactically-aware training, speaker-adaptive prompting, and error-aware evaluation. This study also contributes computational evidence to phonological theory on markedness, sonority, and clarity, which are ripe directions for future work to continue to explore.

Future work can also explore individual variation in ASR performance for under-resourced languages, as well. Understanding individual variation in ASR outcomes has implications beyond linguistic theory. For speakers of under-resourced languages like Tarifit, these insights can inform the development of speech therapy and language learning tools tailored to diverse phonological encoding strategies. Inclusive ASR design also benefits from recognizing how articulatory patterns and perceptual sensitivities vary across users (including those with speech or hearing impairments) supporting broader accessibility goals.

## Conclusion

Our findings show that clear speech significantly improves cross-language ASR transfer to Tarifit, enhancing transcription accuracy and reducing edit distance. This is consistent with prior work on low-resource languages. This effect was robust across phonological patterns: words with rising sonority onset clusters were recognized more accurately than those with falling clusters, and initial geminate consonants were associated with higher error rates, likely due to their acoustic complexity and rarity in Arabic’s phonotactic inventory.

Although individual variability was substantial, the effects of speaking style and sonority were consistent, and word‑level factors accounted for more variance than speaker-level differences. Taken together, our results highlight how phonological structure and speech clarity jointly shape ASR performance. They underscore the need to incorporate typological variation (such as sonority sequencing and gemination) into ASR system design, especially for underrepresented languages. By integrating human-computer interaction (HCI) research with phonological theory, this study demonstrates that communicative adaptations like clear speech offer a naturalistic testbed for evaluating perceptual robustness. The observed disparities in ASR outcomes for marked structures provide empirical support for typological claims linking phonological rarity to perceptual and articulatory difficulty, and point to HCI contexts as fertile ground for advancing both linguistic theory and inclusive technology.

## Data Availability

Data and code available at: https://osf.io/g93kt/overview?view_only=71a7e894afa540379004aede5f2e3f80.
